# *Escherichia coli* Protein Expression System for Acetylcholine Binding Proteins (AChBPs)

**DOI:** 10.1371/journal.pone.0157363

**Published:** 2016-06-15

**Authors:** Nikita Abraham, Blessy Paul, Lotten Ragnarsson, Richard J. Lewis

**Affiliations:** 1 Centre for Pain Research, Institute for Molecular Bioscience, The University of Queensland, St. Lucia, Brisbane, Australia; 2 Division of Molecular Cell Biology, Institute for Molecular Bioscience, The University of Queensland, St. Lucia, Brisbane, Australia; USDA-ARS, UNITED STATES

## Abstract

Nicotinic acetylcholine receptors (nAChR) are ligand gated ion channels, identified as therapeutic targets for a range of human diseases. Drug design for nAChR related disorders is increasingly using structure-based approaches. Many of these structural insights for therapeutic lead development have been obtained from co-crystal structures of nAChR agonists and antagonists with the acetylcholine binding protein (AChBP). AChBP is a water soluble, structural and functional homolog of the extracellular, ligand-binding domain of nAChRs. Currently, AChBPs are recombinantly expressed in eukaryotic expression systems for structural and biophysical studies. Here, we report the establishment of an *Escherichia coli (E*. *coli)* expression system that significantly reduces the cost and time of production compared to the existing expression systems. *E*. *coli* can efficiently express unglycosylated AChBP for crystallography and makes the expression of isotopically labelled forms feasible for NMR. We used a pHUE vector containing an N-terminal His-tagged ubiquitin fusion protein to facilitate AChBP expression in the soluble fractions, and thus avoid the need to recover protein from inclusion bodies. The purified protein yield obtained from the *E*. *coli* expression system is comparable to that obtained from existing AChBP expression systems. *E*. *coli* expressed AChBP bound nAChR agonists and antagonists with affinities matching those previously reported. Thus, the *E*. *coli* expression system significantly simplifies the expression and purification of functional AChBP for structural and biophysical studies.

## Introduction

Nicotinic acetylcholine receptors (nAChR) are promising drug targets for a range of human neurodegenerative diseases [1–6]. Traditionally, drug design and development for nAChR related diseases have mostly followed ligand-based empirical approaches [7–10]. However, nAChR subtypes share conserved features, especially at the orthosteric ligand binding site, necessitating the use of high resolution structural information on receptor-ligand interactions for the rational design of subtype-selective therapeutics [1, 2, 4–6, 11, 12]. Unfortunately, high resolution structures of the nAChRs have been limited by challenges of membrane protein crystallography [9, 12–15]. This challenge has been side-stepped to a significant extent by the use of acetylcholine binding protein (AChBP) as the soluble structural and functional homolog of the nAChR ligand binding domain [13, 16–18]. AChBPs have enabled the application of crystallography and solution based techniques to obtain high resolution insights into nAChR-ligand interactions, required for efficient drug lead optimizations [9, 12, 19, 20].

AChBPs have been recombinantly expressed in eukaryotic expression systems for structural and biophysical studies (Tables 1 and 2). These expression systems offer several advantages, most importantly allowing post-translational modifications that can be important for the solubility and bioactivity of the expressed protein [21, 22]. However, these expression systems are typically more complex, time-consuming and costlier than *E*. *coli* [23–25]. With major advances in the cell lines, expression vectors and fusion proteins available, *E*. *coli* expression systems have become increasingly popular for the production of recombinant proteins [26–28]. Considering that the AChBPs are routinely used for structural and biophysical studies that require milligram quantities of proteins, an *E*. *coli* expression system for the AChBPs would provide an additional, cost-effective and simpler source of protein.

**Table 1:** Comparison of eukaryotic and *E*. *coli* systems for *Lymnaea* (Ls) AChBP expression and purification.

Construct	Expression	Used for	Yield	Purification	Reference
pPIC9 —Ls—AChBP	*Pichia pastoris* GS115	Crystallization	NR	Anion exchange (Poros50 HQ, Mono Q), and gel filtration (Superdex 200)	[14]
pPICZα B—Ls—AChBP	*Pichia pastoris* X-33	Intrinsic tryptophan fluorescence/Crystallization	NR	Anion exchange (Q-Sepharose column)—deglycosylation—Mono Q—gel filtration (Superdex 200)	[29]
pFastbac I—Ls—AChBP—secretion signal sequence	Insect SF9 cells	Crystallization/ITC	NR	Anion exchange (Q-sepharose)—gel filtration (Superdex 200)—Mono Q-sepharose	[30, 31]
p3×FLAG—CMV— 9 —preprotrypsin signal peptide (PPT)— 3xFLAG—Ls-AChBP	HEK cells	Fluorescence Assays	1‒2.5 mg/L	FLAG antibody column	[20]
pHUE—Ls-AChBP	*E*. *coli* (BL21DE3)	Crystallization /Radioligand binding assays	4‒5 mg/L	Affinity (Ni-NTA)—gel filtration (Superdex 200)	This work

NR: not reported.

**Table 2:** Comparison of eukaryotic and *E*. *coli* systems for *Aplysia* (Ac) AChBP expression and purification.

Construct	Expression	Used for	Yield	Purification	Reference
pFastbac I—Ac-AChBP—secretion signal sequence	Insect Sf9 cells	Crystallization / ITC / radioligand binding assays	NR(Secreted protein)	Anion exchange (Q—sepharose)—gel filtration (Superdex 200)—Mono Q-sepharose	[30, 32, 33]
p3×FLAG—CMV— 9 —preprotrypsin signal peptide (PPT)—FLAG—Ac-AChBP	HEK-293S cells	Crystallization	~1‒ 2.5 mg/L	FLAG antibody column FLAG peptide—SEC	[16, 34]
p3×FLAG—CMV— 9 —preprotrypsin signal peptide (PPT)—FLAG—Ac-AChBP— 6x His	HEK-293S cells	Radioligand binding assays	~ 0.5 ‒ 2 mg/L	FLAG antibody column—FLAG peptide—SEC	[16, 34]
pHUE—Ac-AChBP	*E*. *coli* (BL21DE3)	Radioligand binding assays	1‒ 1.5 mg/L	Affinity (Ni—NTA)—gel filtration (Superdex 200)	This work

NR: not reported.

Furthermore, attempts have been made to use NMR techniques to monitor ligand induced conformational changes of the nAChR in solution. Isotopically labelled (^15^N and ^13^C) ligand binding domains of bacterial homologs have been expressed for this purpose, but these failed to oligomerize as a pentamer in solution and therefore lacked an intact ligand binding pocket [35, 36]. AChBP naturally assembles as a pentamer in solution and therefore is a suitable candidate for such studies. AChBPs with isotopically labelled cysteine residues have demonstrated conformational changes of the C-loop associated with ligand binding [37]. However, this selected residue labelling does not allow the identification of global conformational changes that link ligand binding to channel gating. An *E*. *coli* expression system expressing AChBP would be ideal for isotopic labelling required for the NMR analysis of large protein molecules. Previous attempts to express the AChBPs in *E*. *coli* produced only insoluble AChBP, requiring expensive and time-consuming stabilization and refolding steps to obtain functional protein [38].

In this study, we report a high-throughput and economical *E*. *coli* expression system capable of generating stable, soluble and functional AChBPs from *Lymnaea stagnalis* (Ls) and *Aplysia californica* (Ac), which are the most widely used AChBPs. We achieved this by tagging the AChBPs with ubiquitin (Ub), which enhanced the expression of correctly folded AChBPs in the soluble fractions. Optimized expression parameters produced milligram quantities of fusion protein per liter of culture that was readily enzymatically de-ubiquitinylated to yield purified, native AChBPs for structural and biophysical applications.

## Materials and Methods

### Construct design

Synthetic Ls- and Ac-AChBP genes were designed and purchased (Blue Heron Biotechnology). The AChBP genes were PCR-amplified from pUC19 cloning vector using primers with SacII and NotI restriction sites, respectively, for cloning into the pHUE vector [39] for bacterial expression. The resulting construct included the hexa-histidine tag for detection and purification, followed by genes for ubiquitin and Ls/Ac AChBP respectively. (S1 Fig). All cloning work was carried out using the One Shot^®^ Top10 chemically competent *E*. *coli* cells (Life Technologies) and clones were confirmed by sequencing by the Australian Genome Research Facility.

### Optimization of *E*. *coli* protein expression

Protein expression conditions (Table 3) for Ls-AChBP and Ac-AChBP were optimized using small scale (10 mL) cultures. Plasmids encoding the AChBPs were transformed into *E*. *coli* BL21DE3 cells and plated onto Luria Bertani (LB) agar with ampicillin (Sigma-Aldrich) for selection (0.1 mg/mL). Following overnight incubation, a single colony was picked and inoculated into 5 mL LB with ampicillin (0.1 mg/mL), and incubated overnight at 37°C with constant shaking. The following day 250 μL of the overnight cultures were inoculated into 10 mL of LB, with ampicillin (0.1 mg/mL). For routine protein expression, overnight cultures were also stored as glycerol stocks by mixing 500 μL of culture with 500 μL sterile 60% glycerol at ‒80°C. Cells were grown under appropriate expression conditions (Table 3) and harvested using high speed centrifugation (17,000 x g). Cell pellets were re-suspended in, 20 mM Tris, 150 mM NaCl, 10% glycerol, pH 8.0 (AChBP) and 20 mM HEPES, 300 mM NaCl, pH 7.0 (DUB) to a uniform OD_600_ to ensure equal protein loading. Cells were lysed using sonication and the cell debris (insoluble/inclusion body material) was separated from the soluble material using high-speed centrifugation. Soluble and insoluble fractions were analyzed using western blots. Protein bands were quantified by densitometric analysis (Odyssey, Licor^®^ Biosciences), where intensity was measured by defining each band with the rectangular tool.

**Table 3:** Parameters optimized for the soluble expression of AChBPs.

Parameters	Range
Temperature	37°C, 25°C, **16°C**
Expression induction method	**IPTG** and Auto induction
Inducer concentration *(only for Ac-AChBP)*	1 mM and **0.1 mM**
Cell density for induction i.e O.D_600_ *(only for Ac-AChBP)*	0.8, 0.5, **0.1**.

Optimal expression conditions are indicated in bold.

### Large-scale expression of AChBP in *E*. *coli*

Large scale protein production for Ls- and Ac-AChBPs were carried out in 2–6 L of 2x Yeast extract Tryptone (2xYT) media using the optimized expression conditions. Expression was performed in 2xYT media, which is capable of supporting higher biomass than LB and therefore improves protein expression levels. Protein expression was induced using 1 mM IPTG at 16°C and 0.5 OD_600_ for Ls-AChBP and DUB. For Ac-AChBP, expression was induced with 0.1 mM IPTG at 16°C and 0.2 OD_600_. Cells were harvested by centrifugation (6,076 x g) and the cell pellet re-suspended in 20 mM Tris, 150 mM NaCl, 10% glycerol, pH 8.0 for AChBPs and 20 mM HEPES, 300 mM NaCl, pH 7.0 for DUB. Cell pellets were stored at –80°C until used for purification.

### Expression of AChBP in minimal media

The protein expression method followed here is adapted from Marley *et al*., 2010 and Shivashanmugam *et al*. [40, 41]. The pHUE expression construct for Ls-AChBP transformed into BL21DE3 cells were used for the expression. Cells were grown in LB broth to an O.D of 0.7–0.8 after which they were pelleted at 5,000 x g for 30 min. The pellets were gently re-suspended in 1 x M9 salts (22 mM KH_2_PO_4_, 90 mM Na_2_HPO_4_, 17 mM NaCl) to remove residual LB broth. The re-suspended cells were again pelleted at 5,000 x g for 30 min, re-suspended in 1L of minimal media (1 x M9 salts 20% (w/v),Vitamin solution 0.2% (w/v),Thiamine solution 0.2% (w/v),1 M MgSO_4_ 0.16% (w/v), 1 M CaCl_2_ 0.008% (w/v), ^15^NH_4_Cl 0.1% (w/v), ^13^C D-glucose 0.4% (w/v), Ampicillin 0.1 mg/mL) and grown at 37°C for 1–1.5 hours before protein expression was induced with 1 mM IPTG (final concentration) at 16°C for 18–20 h. Finally, cells were harvested at 5,000 x g for 30 min at 4°C, the pellet re-suspended in 20 mM Tris, 150 mM NaCl, 10% glycerol and pH 8.0 and stored at –80°C prior to use.

### Large-scale purification of AChBP

Large-scale purification was carried out using a two-step purification protocol combining immobilized metal affinity chromatography (IMAC) and size exclusion chromatography (SEC). Cell pellets were lysed by repeated (3 x) freeze-thawing of the pellets, sonication and incubation with lysozyme at a final concentration of 0.5 mg/mL (Sigma-Aldrich). To reduce viscosity and to prevent proteolysis of the expressed protein DNAase, (10 units/μL) (Roche) and protease inhibitor tablets (1 tablet/50 mL of supernatant, cOmplete EDTA-Free Roche) were added. The lysed cells were pelleted at 39,000 x g and the supernatant purified by IMAC using HIS-select^(R)^ HF nickel affinity gel (Sigma Aldrich). To assess homogeneity and oligomerization state, the IMAC purified AChBPs were analyzed on an analytical grade Superdex 200 (S200) 10/300 column (GE healthcare) calibrated with molecular weight standards (blue dextran (2,000,000 Da), β-amylase (200,000 Da), alcohol dehydrogenase (150,000 Da), albumin (66,000 Da), carbonic anhydrase (29,000 Da), and cytochrome C (12,400 Da) from Sigma-Alrich) on an ÄKTA FPLC system (GE healthcare). Theoretical molecular weights for AChBPs were calculated using expasy Protparam [42].

### Cloning, expression and purification of DUB

Deubiquitinylating enzyme (DUB) was used in this study to cleave the AChBP-Ub fusion protein to yield native AChBPs. The gene for DUB cloned into the pET15b vector was kindly provided by Prof. Bostjan Kobe. Screening for optimal *E*. *coli* expression conditions and purification of DUB were performed using the same methods employed for AChBP, as described above.

### Ubiquitin cleavage

The AChBPs were cleaved from ubiquitin using the DUB enzyme incubated in a 1:50 (enzyme:protein) ratio at 4°C for ~18 h at pH 8.0, as reported previously [39]. The cleaved proteins were then purified using a pre-equilibrated S200 16/600 SEC column (GE healthcare). The fractions containing the protein were pooled and concentrated to the desired concentration using an ultrafilter (Amicon Ultra-15 MWCO 10kDa, Merck Millipore). For AChBP expressed in minimal media, an additional buffer exchange step was introduced after concentrating the sample and the labelled protein was transferred to a buffer more suitable for NMR (20 mM Tris, 100 mM NaCl and pH 7.5) using dialysis.

### Radioligand binding assay at AChBP

Competitive radioligand binding assay with ^3^H-epibatidine (specific activity 1.11–2.59 TBq/mmol) and nAChR agonists and antagonists was used to determine the functional activity of the recombinant Ls- and Ac-AChBPs. The proteins were coated onto 96 well plates (Flexible PET Microplate, Perkin Elmer) at a concentration of 8 μM total protein. Serial dilutions of nAChR ligands together with a fixed concentration of ^3^H-epibatidine (1 nM) were incubated with the immobilized protein in 100 μL of assay buffer (phosphate buffered saline with 0.05% bovine serum albumin) for 1 h at 4°C. Unbound ligands were washed manually followed by addition of 100 μL scintillant (Optiphase Supermix, Perkin Elmer). Plates were incubated with the scintillant for 2 min on a shaker and radioactivity measured with a Wallac 1450 MicroBeta liquid scintillation counter. Binding data were evaluated by a nonlinear, least squares one-site competition fitting procedure using GraphPad Prism 6.0 (GraphPad Software Inc., San Diego, CA, USA). The radioligand binding assays were performed in triplicate in three separate experiments (*n* = 3), with IC_50_ values reported as mean ± standard error of the mean (S.E.M).

## Results

### Screening of protein expression conditions

**Fig 1:**
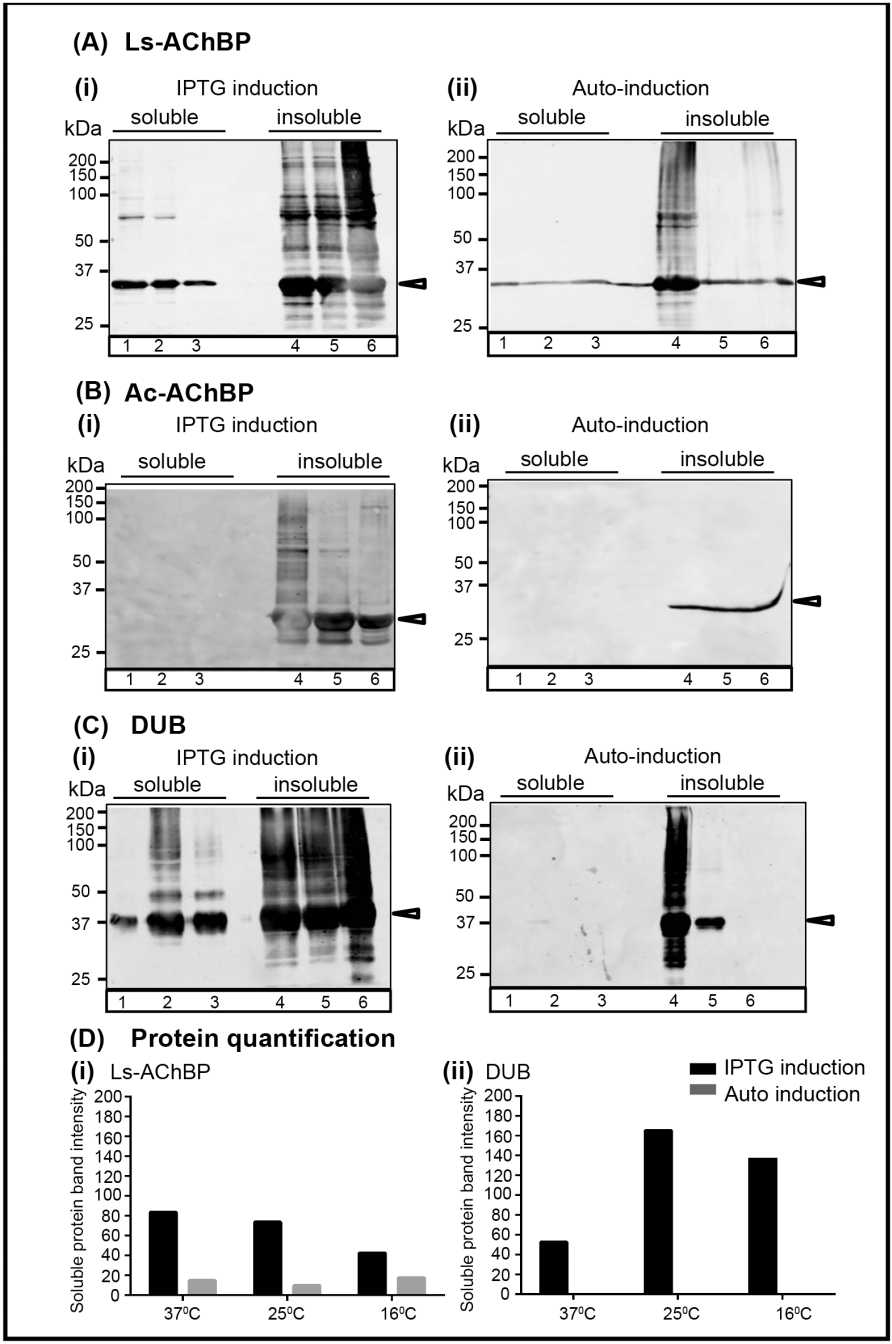
Expression optimization of AChBP and DUB in *E*. *coli*. Protein expression levels in the soluble fractions at (1) 37°C, (2) 25°C and (3) 16°C and insoluble fractions (inclusion body) at (4) 37°C, (5) 25°C and (6) 16°C using the (i) IPTG and (ii) auto induction methods were analyzed for **(A)** Ls-AChBP, **(B)** Ac-AChBP and **(C)** DUB using western blots. Anti-His tag mouse monoclonal antibody at a dilution of 1:3000 in the blocking solution was used as the primary antibody Fluorescently labelled goat anti-mouse IgG (H+L) Alexa Fluor 680 at a dilution of 1:2500 was used as the secondary antibody. The western blots were imaged with an Odyssey Infrared Imaging System (Licor^®^ Biosciences) at a wavelength of 700 nm and 2.0 intensity level. Black arrows indicate expected bands for AChBPs and DUB. **(D)** Protein expression level in the soluble fraction under the different induction methods and temperatures were quantified for (i) Ls-AChBP and (ii) DUB. Ac-AChBP was not detected in the soluble fraction and therefore not quantified.

AChBP and DUB expression levels were evaluated at different temperatures and induction methods to determine the optimum parameters for protein expression (Table 3). Ls-AChBP was successfully expressed in the soluble fraction using both IPTG and auto induction methods. Optimum expression levels were observed with 1 mM IPTG induction at 37°C (Fig 1A). However, Ls-AChBP expressed at lower temperature (16°C) was more stable during purification, hence routine expression of Ls-AChBP was performed at 16°C. In contrast, expression of Ac-AChBP was detected in the insoluble fraction of both the auto induced and IPTG induced cultures (Fig 1B). Therefore, the rate of protein expression was lowered by reducing the inducer concentration and the optical density (OD_600_) of the culture at which protein expression is induced. This was based on a previous report suggesting that high rate of protein expression often leads to accumulation of the recombinant protein in inclusion bodies [43]. Using a low concentration of IPTG (0.1 mM) and low optical density of the culture (OD_600_: 0.2) Ac-AChBP was expressed in the soluble fraction, albeit at lower concentrations (final purified yield: 1–3 mg/L) compared to that for Ls-AChBP (final purified yield: 4–5 mg/L). IPTG induced expression of DUB was superior to auto induction, with higher protein expression levels at 16°C compared to the previously reported 37°C [39]. Therefore, routine expression of DUB was carried out with IPTG induction (1 mM) at 16°C (Fig 1C).

### Recombinant AChBPs are soluble and pentameric in solution

**Fig 2:**
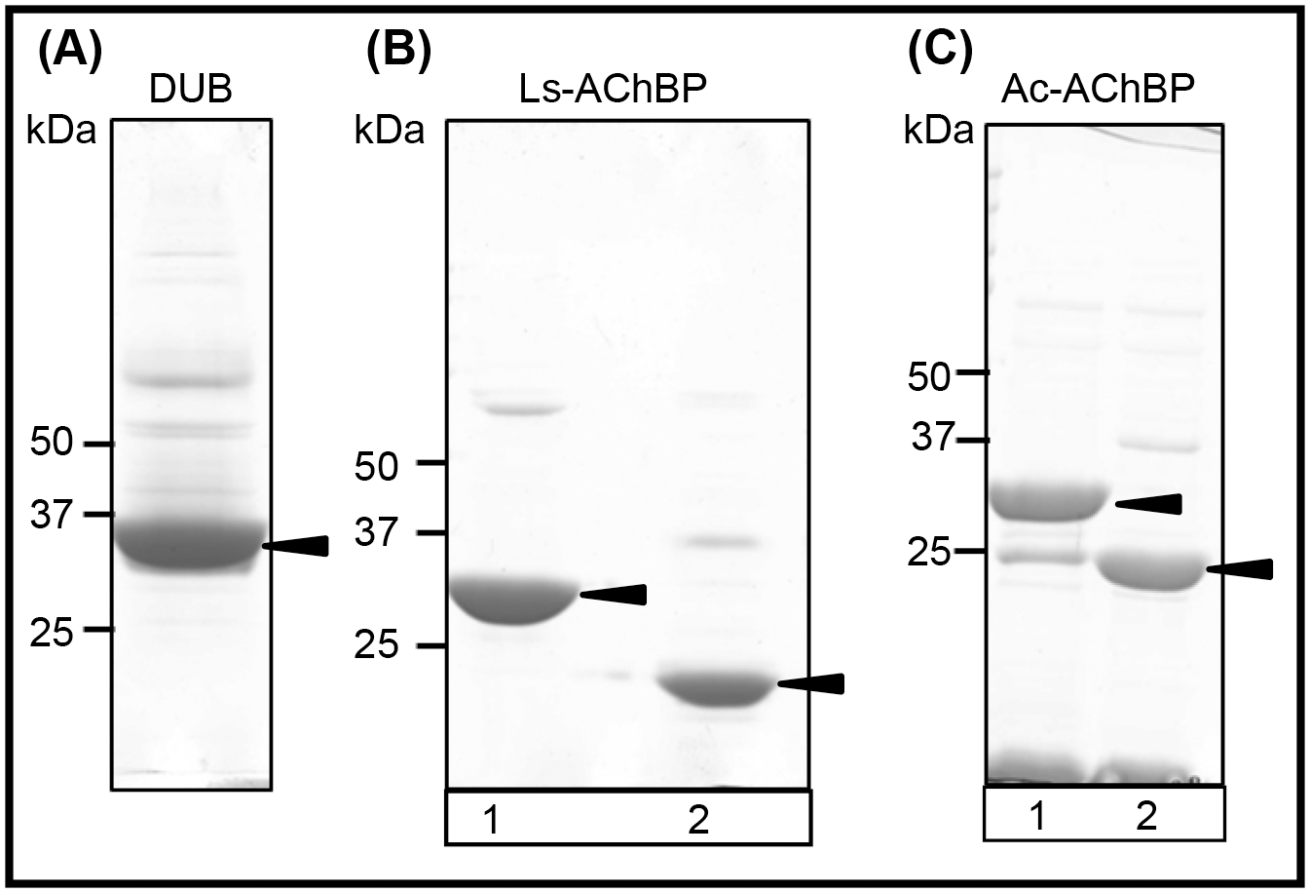
IMAC purified Ls-, Ac-AChBP and DUB. **(A)** Recombinantly expressed DUB migrates as the major band to a relative molecular weight of 39.7 kDa. (**B)** Lane 1: IMAC purified ubiquitin tagged Ls-AChBP with a relative molecular weight of 30.2 kDa. Lane 2: De-tagged Ls-AChBP migrates to a relative molecular weight of 19.2 kDa. (**C)** Lane 1: IMAC purified ubiquitin tagged Ac-AChBP with a relative molecular weight of 35.9 kDa. Lane 2: De-tagged Ac-AChBP migrates to a relative molecular weight of 24.6 kDa. SDS-page gels were stained Coomassie Brilliant Blue R250 and imaged with ImageScanner III (GE healthcare) at a resolution of 600 dpi.

IMAC purified DUB had a relative molecular weight of 39.7 kDa (based on calculated relative migration distance on the SDS-PAGE gel). DUB cleaved ubiquitin from the AChBPs, as observed from the difference in the relative molecular weights for the AChBPs before and after incubation with DUB (Fig 2B and 2C). The tagged Ls- and Ac-AChBP were found to have a relative molecular weight of 30.2 kDa and 35.9 kDa, respectively. De-tagged Ls- and Ac-AChBPs were found to have a molecular weight of 19.2 and 24.6 kDa, a difference of ~11 kDa, corresponding to the molecular weight of ubiquitin (10.3 kDa.)

**Fig 3:**
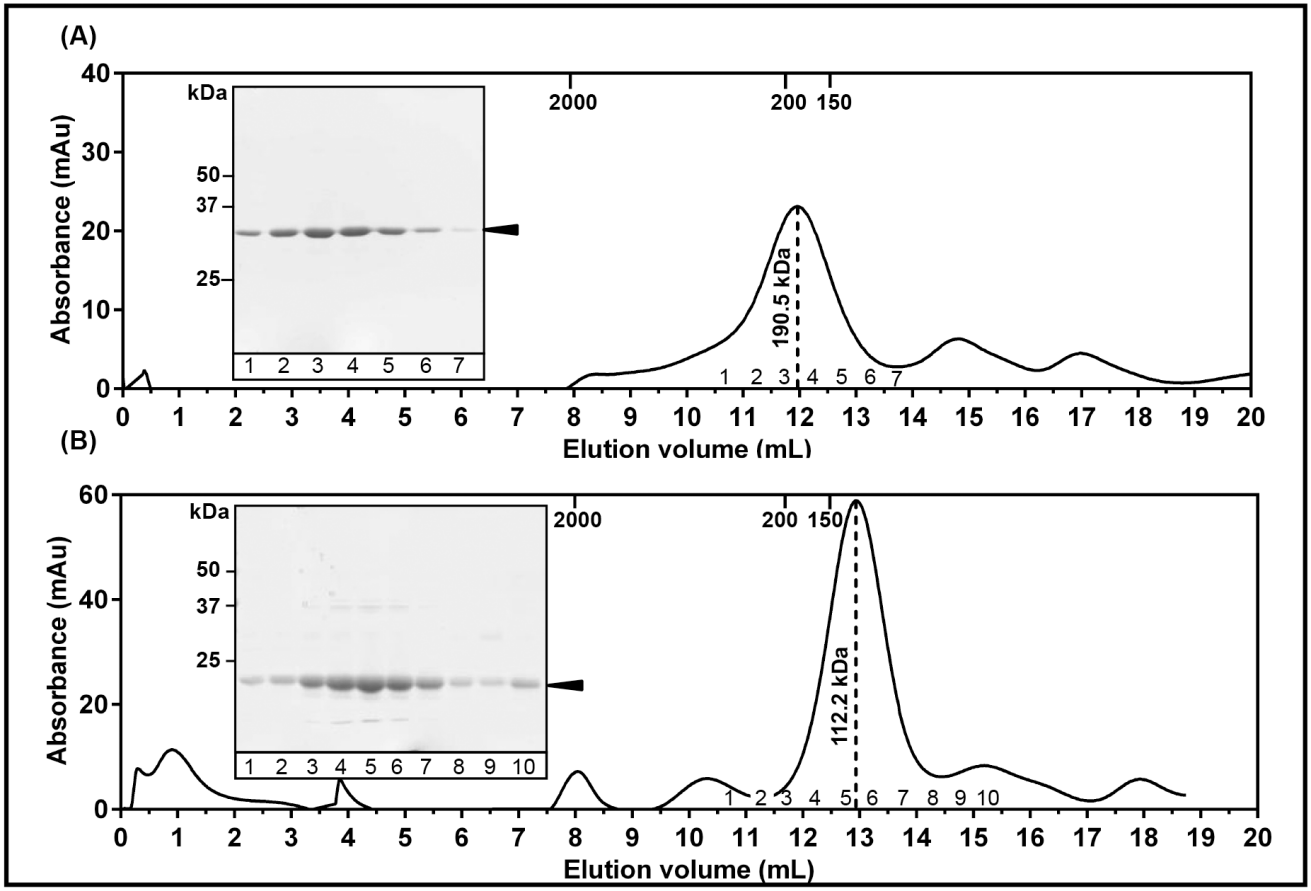
Ls-AChBP is pentameric in solution. Elution profile on a calibrated analytical size exclusion chromatography column (S200 10/300) for (**A)** pentameric, ubiquitin tagged Ls-AChBP with a molecular weight of 190.5 kDa. Peak fractions were analyzed on a 10% SDS-PAGE gel (inset) and **(B)** pentameric, de-tagged Ls-AChBP with a molecular weight of 112.2 kDa. Peak fractions were analyzed on a 12% SDS-PAGE gel (inset). Elution volumes for standard proteins used for calibration are indicated on top of each panel.

**Fig 4:**
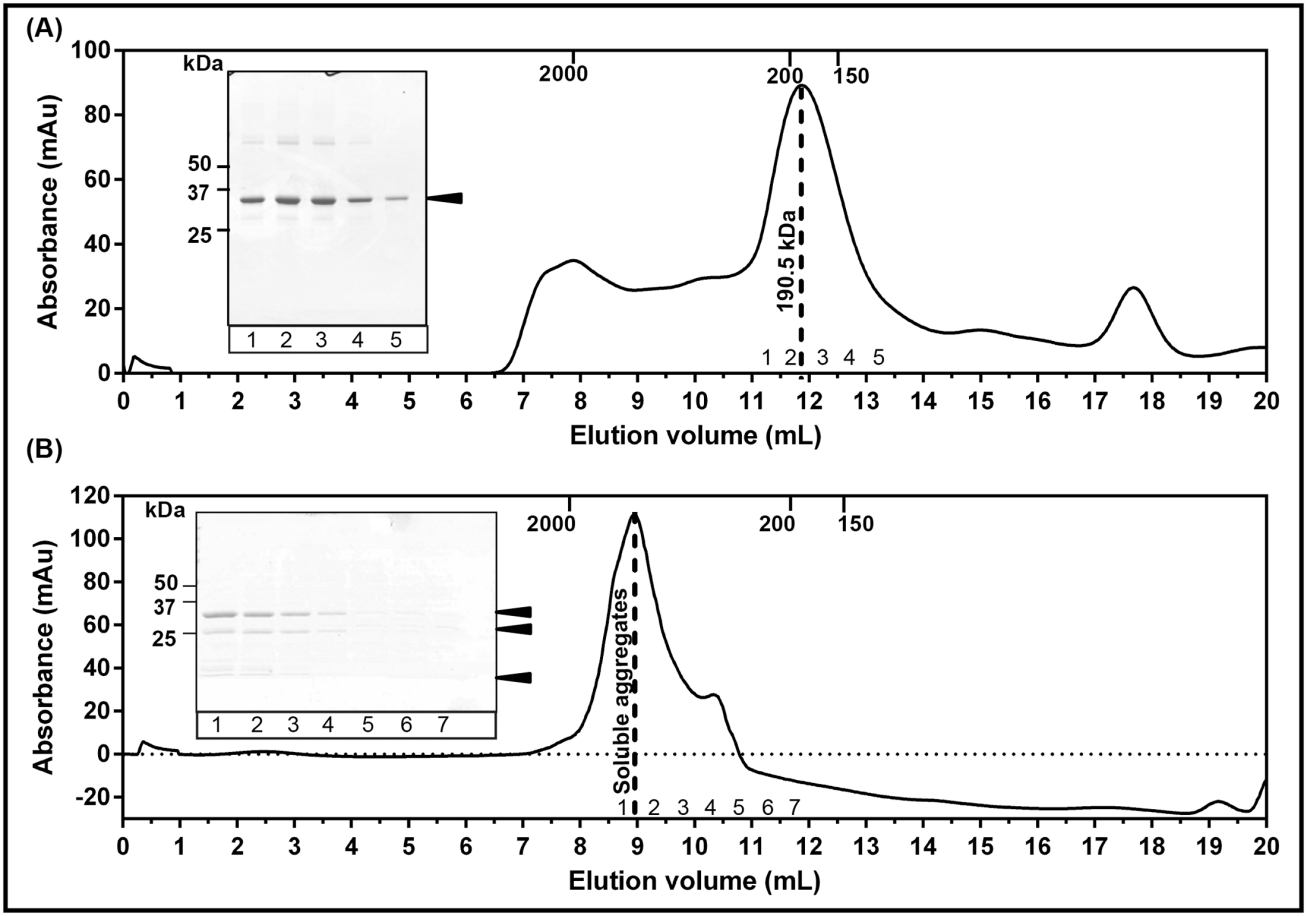
Ac-AChBP is pentameric in solution. Elution profile on a calibrated analytical size exclusion chromatography column (S200 10/300) for (**A)** pentameric, ubiquitin tagged Ac-AChBP with a molecular weight of 190.5 kDa. Peak fractions were analyzed on a 10% SDS-PAGE gel (inset) and **(B)** pentameric, de-tagged Ac-AChBP eluted at void volume as soluble aggregates. Peak fractions were analyzed on a 12% SDS-PAGE gel (inset). Elution volumes for standard proteins used for calibration are indicated on top of each panel.

**Fig 5:**
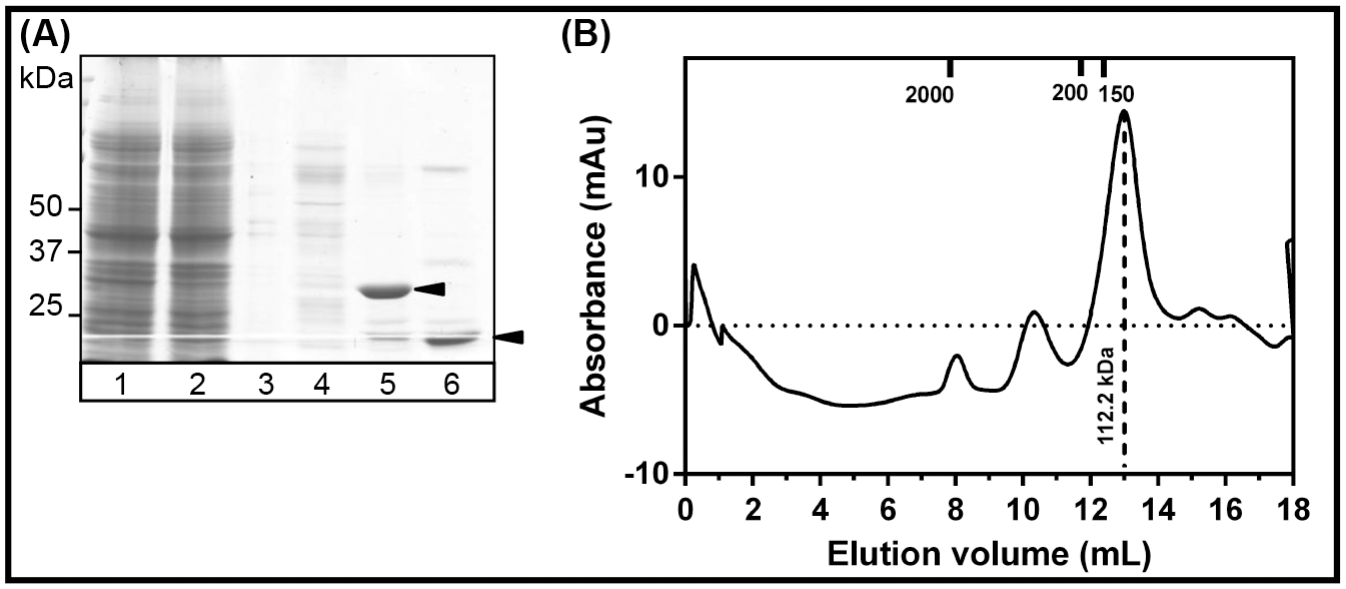
Ls-AChBP expressed in minimal media. (**A)** Ls-AChBP expressed in minimal media with ^15^NH_4_Cl at different stages of purification, (1) lysate (2) unbound proteins (3, 4) washes (5) ubiquitin tagged Ls-AChBP (6) de-tagged Ls-AChBP. (**B)** Size-exclusion chromatogram for de-tagged, Ls-AChBP eluting as a pentamer with an experimental molecular weight of 112.2 kDa.

Purified AChBPs were analyzed on a calibrated analytical Superdex 200 column (GL10/300) to assess homogeneity and oligomerization state in solution. The ubiquitin tagged AChBPs eluted as a major, homogenous peak at an elution volume consistent with the molecular weight for pentameric, ubiquitin-tagged AChBPs (Figs 3A and 4A, S5 Fig and S1 Table). De-tagged Ls-AChBP remained a soluble pentamer after cleavage of the ubiquitin (Fig 3B). However, de-tagged Ac-AChBP was found to be unstable in solution without the ubiquitin tag (Fig 4B). Therefore, ubiquitin was not cleaved from Ac-AChBP and the tagged protein can be used in studies not affected by presence of fusion partners. Fractions corresponding to the protein peak were found to be >90% pure when analyzed on SDS-PAGE gels, and therefore suitable for functional as well as structural studies requiring highly purified protein samples. The final yield of purified proteins was found to be 4–5 mg/L for Ls-AChBP and 1–1.5 mg/L for Ac-AChBP. Similarly, Ls-AChBP expressed in minimal media was found to be stable and pentameric in solution with an elution profile matching that of Ls-AChBP expressed in 2xYT (Fig 5). The final yield for Ls-AChBP purified from minimal media was 1–1.5 mg/L of expression, about 5-fold lower than that obtained from expression in 2xYT media.

### Recombinant Ls and Ac-AChBPs are functional

**Fig 6:**
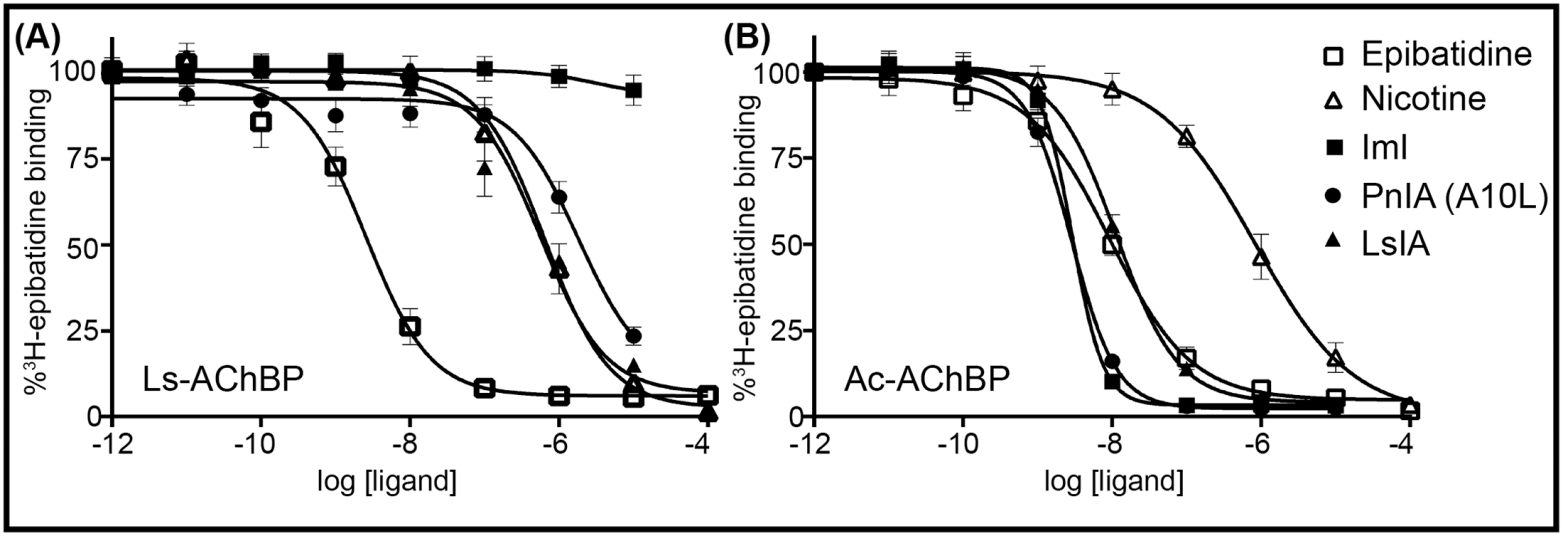
AChBPs expressed in *E*. *coli* are functional. Competitive radioligand binding assay with [^3^H]-epibatidine and nAChR ligands were used to determine the activity of (**A)** Ls-AChBP and (**B)** Ac-AChBP.

To confirm that recombinant AChBPs expressed in *E*. *coli* cultures were functional, their ability to interact with nAChR ligands was tested (Tables 4 and 5, Fig 6) in a competitive radioligand binding assay. Both AChBPs had affinities for epibatidine, nicotine, ImI, PnIA (A10L) and LsIA that were similar to literature values (Tables 4 and 5). Both AChBPs displayed low nano molar affinities for epibatidine and micro molar affinities for nicotine. Ac-AChBP was found to have nano molar affinities for the α-conotoxins, whereas Ls-AChBP displayed micro molar affinities for these peptide antagonists, a typical binding behavior observed in previous studies [32, 44]. This confirms that the AChBPs expressed in this *E*. *coli* expression system have similar binding properties as that of AChBPs expressed in eukaryotic expression systems.

**Table 4:** Binding properties of Ac-AChBP expressed in *E*. *coli*.

Ligand	K_i_ ± S.E.M (nM) (This work)	Literature K_i_ (nM)	Mode of action
Epibatidine	11.57 ± 4.8	11.4 ± 0.7 [45]	Agonist
Nicotine	1056 ± 640	583 ± 84 [45]	Agonist
ImI	3.0 ± 0.5	4.0 ± 2 [45]	Antagonist
PnIA (A10L)	2.8 ± 0.2	36.7 ± 16.6 [32]	Antagonist
LsIA	11.0 ± 0.8	ND	Antagonist

Binding constants (K_i_ values) were determined using competitive radioligand binding assay with ^3^H-epibatidine (*n* = 3) each performed in triplicate. ND, not determined.

**Table 5:** Binding properties of Ls-AChBP expressed in *E*. *coli*.

Ligand	K_i_ ±S.E.M (nM) (This work)	Literature K_i_ (nM)	Mode of action
Epibatidine	3.5 ± 1.6	2.5 ± 0.2 [45]	Agonist
Nicotine	860 ± 400	1100 ± 230 [45]	Agonist
ImI	>10,000	>10,000 [45]	Antagonist
PnIA (A10L)	2000 ± 1100	80 ± 30 [32]	Antagonist
LsIA	700 ± 370	ND	Antagonist

Binding constants (K_i_ values) were determined using competitive radioligand binding assay with ^3^H-epibatidine (*n* = 3), each performed in triplicate. ND, not determined.

## Discussion

AChBPs are widely used structural templates for the extracellular ligand binding domain of the nAChRs [19]. Currently, AChBPs are expressed in eukaryotic systems, which are more suitable than *E*. *coli* systems for large proteins such as AChBP, which also have post-translational modifications like N-linked glycosylation [14, 16, 29–34, 46]. Subsequently, glycosylation was shown not to be important for binding properties of the protein. In fact, de-glycosylation of the protein and/or a more homogenous glycosylation pattern was required to facilitate crystallization of the protein [30, 34]. This presented an opportunity to develop an additional recombinant expression system in *E*. *coli*, which would be a faster, simpler and economical source of this protein for biophysical studies. However, previous attempts of AChBP expression in *E*. *coli* resulted in accumulation of these proteins in inclusion bodies [38]. This necessitated the use of expensive and time-consuming re-solubilization techniques to recover the expressed protein.

In this study we have established an *E*. *coli* expression system capable of expressing AChBPs in the soluble fractions. We successfully expressed Ls- and Ac-AChBPs using this expression system. Our method overcomes three major challenges (1) absence of post-translational machinery prevents protein-glycosylation that can interfere with crystallization, (2) AChBPs are expressed in the soluble fractions, therefore overcoming the need to recover protein from inclusion bodies and (3) the *E*. *coli* expression system reduces the complexity and cost of production by almost 10-fold compared to eukaryotic expression systems. It significantly reduces the large scale protein expression and purification time frame from 2–3 weeks using eukaryotic expression systems, to about 3–4 days. Additionally, we report optimized protein expression conditions capable of generating milligram quantities of AChBPs. Yield and quality of the AChBPs expressed in *E*. *coli* match that of AChBPs expressed from existing AChBP expression systems and meet the standards required for techniques such as x-ray crystallography.

Expression of AChBPs in the soluble fractions was largely facilitated by the use of ubiquitin (Ub) which serves as a fusion tag. *E*. *coli* lacks Ub and all enzymes in the ubiquitin pathway, thus preventing any non-specific proteolysis [39, 47, 48]. Previously, Ub has been particularly valuable in driving the soluble expression for several difficult to express proteins [48]. Availability of de-ubiquitinylating enzymes (DUB) provides a convenient method to cleave Ub from protein of interest efficiently [47]. These enzymes recognize the double glycine motif at the C-terminal of Ub, ensuring specific cleavage without leaving additional residues, unlike several commonly used proteases [49]. Additionally, DUBs can be expressed in-house, using *E*. *coli*, further reducing overall expression costs for routinely expressed proteins such as AChBPs [39].

Hexa-His tagged Ub incorporated in the pHUE expression vector [39] was used in this study for the *E*. *coli* expression of AChBPs. Using this construct, sufficient quantities of AChBPs could be successfully retrieved from the soluble fraction; therefore solubilization of AChBPs accumulated in insoluble fractions was not required. In addition, both AChBPs spontaneously oligomerize in the native pentameric form. Further, we were able to successfully remove the ubiquitin from Ls-AChBP, and de-tagged Ls-AChBP retained the pentameric oligomerization state. A range of crystallization conditions yielded diffracting crystals for de-tagged Ls-AChBP providing diffraction data up to 2.8 Å (S4 Fig), confirming that the *E*. *coli* expression system does not compromise AChBP crystallization. On the contrary, de-tagged Ac-AChBP was seen to aggregate in solution. Ubiquitin tagged Ac-AChBP could potentially pose a problem for crystallographic studies, although some examples in the literature suggest that fusion tags could potentially improve crystallization of the protein [50]. However, it remains to be seen whether the ubiquitin allows or hinders the crystallization of Ac-AChBP. Importantly, the binding properties of AChBPs generated using this system were not altered, with the pharmacological profiles of both Ls- and Ac-AChBP matching previously reported values. As expected, Ac-AChBP exhibited higher affinity for the nAChR agonists and antagonists compared to Ls-AChBP, consistent with the observed trend [32, 44, 51].

Previous attempts have been made to express isotopically-labelled ligand binding domains of nAChR homologs to monitor conformational changes in solution [35, 37]. However, these are limited by the absence of an intact ligand binding pocket as is the case for *gleobacter violaceus* ligand-gated ion channel which remains a monomer in solution [35]. AChBP overcomes this limitation and not only exists as a pentamer in solution, but has been shown to possess channel gating properties, therefore providing a reliable template to observe ligand-associated channel gating mechanisms [52]. To facilitate NMR studies with AChBP, we utilized the *E*. *coli* expression system to express Ls-AChBP in minimal media. Although successful use of AChBP in NMR will require further optimization, including buffer conditions and deuteration, we demonstrated that it is possible to produce isotopically labelled AChBP that could allow monitoring of dynamic processes such as ligand-induced conformational changes linked to channel gating.

Recently, important breakthroughs have been made in determining the structure of the ligand binding domain of the nAChR [53]. However, these structures do not have an intact ligand-binding pocket, and therefore do not allow characterization of subtype-selective receptor and orthosteric ligand interactions. In the absence of high resolution structures of the nAChR extracellular domain, AChBPs are the only nAChR homologs that exist in a soluble, pentameric form facilitating biophysical characterization of nAChR-ligand interactions. AChBPs are now being adapted through mutagenesis to mimic nAChR and other Cys-loop ligand binding pockets including the 5-HT3 receptor [54, 55]. *E*. *coli* expression provides an economical, and simpler alternative to eukaryotic systems currently used for the production of AChBPs, facilitating more comprehensive structural studies including the structure-based drug design of nAChR specific therapeutics.

## Supporting Information

S1 Fig pHUE vector map and Ls/Ac-AChBP expression construct. (PDF)

S2 Fig Ls-AChBP DNA and protein sequences. (PDF)

S3 Fig Ac-AChBP DNA and protein sequences. (PDF)

S4 Fig Crystallization of *E*. *coli* expressed Ls-AChBP. (PDF)

S5 Fig Calibration curve used to estimate molecular weights for AChBPs. (PDF)

S1 Table Experimental and theoretical molecular weights. (PDF)
