# Supplementary material for: Escherichia coli Protein Expression System for Acetylcholine Binding Proteins (AChBPs)
Source: PLoS One. 2016 Jun 15;11(6):e0157363. doi: 10.1371/journal.pone.0157363 (PMC4909209; doi:10.1371/journal.pone.0157363)
Supplement: S1 Fig — (PDF) [file pone.0157363.s001.pdf]

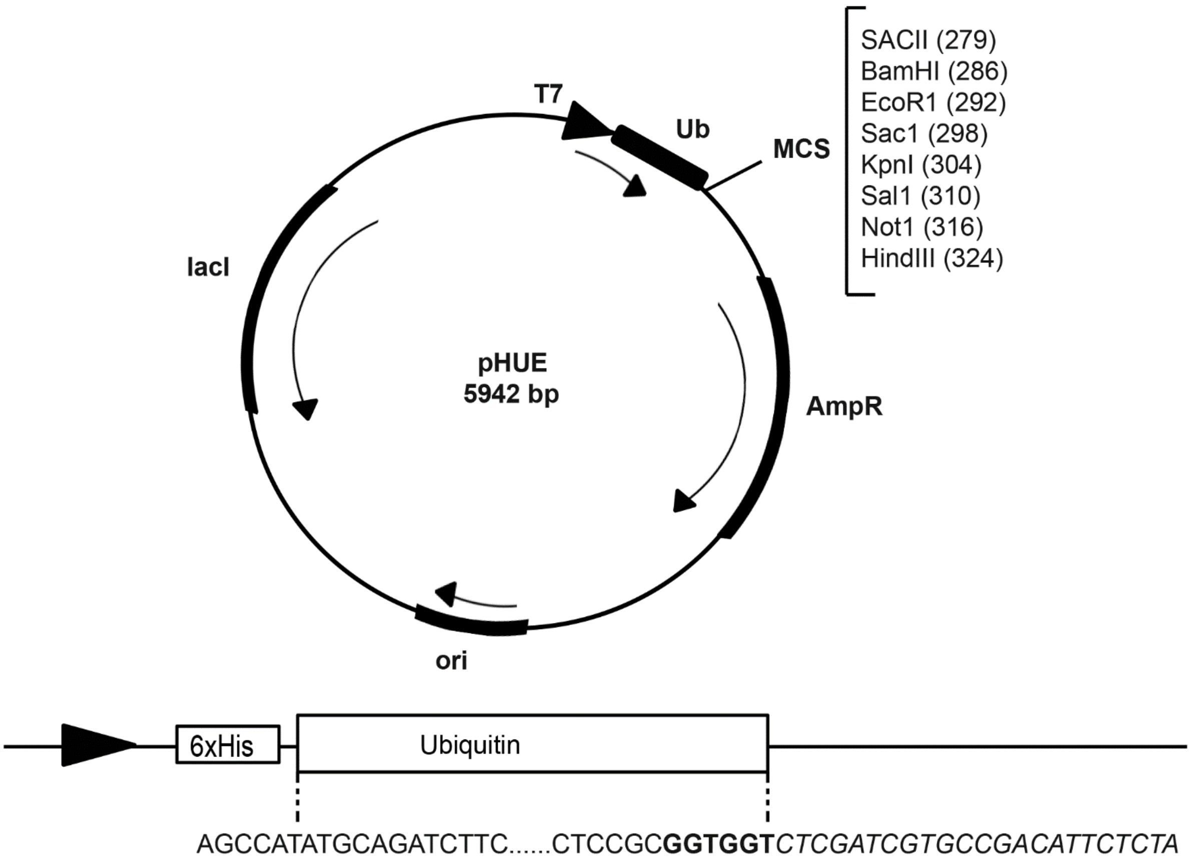

S1 Fig. pHUE vector map and Ls/Ac-AChBP expression construct. Protein expression is under the control of a T7 promoter and uses ampicillin resistance for selection. Ubiquitin (Ub) serves as the fusion partner, allowing soluble expression of the target protein. The 6xHis assists in the purification of the fusion proteins. The construct used in this study consists of the DUB recognition sequence (bold) at the ubiquitin C-terminal followed by the Ls/Ac-AChBP sequence (italics).
