# Supplementary material for: Escherichia coli Protein Expression System for Acetylcholine Binding Proteins (AChBPs)
Source: PLoS One. 2016 Jun 15;11(6):e0157363. doi: 10.1371/journal.pone.0157363 (PMC4909209; doi:10.1371/journal.pone.0157363)
Supplement: S2 Fig — (PDF) [file pone.0157363.s002.pdf]

(A)

```
        10         20         30         40         50         60         70         80
TTGGACCGGG CAGACATCTT GTACAACATA CGTCAGACAT CGAGACCGGA TGTGATTCCC ACACAGCGAG ATCGCCCAGT
        90        100        110        120        130        140        150        160
GGCGGTGTCC GTCTCTTTGA AGTTCATCAA CATCTTGGAA GTGAATGAAA TAACCAATGA AGTGGACGTG GTCTTTTGGC
       170        180        190        200        210        220        230        240
AGCAGACGAC ATGGTCGGAC AGGACCCTCG CCTGGAACAG TTCTCACTCA CCAGATCAGG TTTCCGTGCC AATAAGCTCT
       250        260        270        280        290        300        310        320
TTGTGGGTGC CTGACCTCGC TGCATACAAC GCCATCTCGA AACCTGAAGT CCTTACACCG CAACTGGCCA GGGTCGTATC
       330        340        350        360        370        380        390        400
CGATGGTGAA GTGCTGTACA TGCCGAGTAT CCGCCAGCGG TTCTCCTGCG ATGTATCGGG TGTCGATACG GAGTCCGGTG
       410        420        430        440        450        460        470        480
CTACATGTCG GATCAAAATT GGTTCCTGGA CCCACCACAG TAGAGAGATT TCTGTAGATC CCACGACAGA AAATAGTGAT
       490        500        510        520        530        540        550        560
GATTCTGAAT ACTTCTCCCA ATACTCTCGC TTTGAAATCT TGGACGTCAC ACAGAAGAAG AACTCGGTTA CCTACTCTTG
       570        580        590        600        610        620        630        633
CTGTCCGGAG GCATACGAGG ACGTTGAAGT GAGTCTCAAT TTCCGGAAGA AGGGACGCTC CGAAATTCTT TAG
```

(B)

LDRADILYNIRQTSRPDVIPTQRDRPVAVSVSLKFINILEVNEITNEVDVVFWQQTTWSDRTLAWNSSHSPDQVSVPISSLWV
PDLAAYNAISKPEVLTPQLARVVSDGEVLYMPSIRQRFSCDVSGVDTESGATCRIKIGSWTHHSREISVDPTTENSDDSEYFS
QYSRFEILDVTQKKNSVTYSCCPEAYEDVEVSLNFRKKGRSEIL

S2 Fig. Ls-AChBP DNA and protein sequences. (A) Ls-AChBP (mammalian) DNA sequence (B) Protein sequence used.
