# Supplementary material for: Escherichia coli Protein Expression System for Acetylcholine Binding Proteins (AChBPs)
Source: PLoS One. 2016 Jun 15;11(6):e0157363. doi: 10.1371/journal.pone.0157363 (PMC4909209; doi:10.1371/journal.pone.0157363)
Supplement: S3 Fig — (PDF) [file pone.0157363.s003.pdf]

(A)

```
 10         20         30         40         50         60         70         80
CACAGCCAAG CAAACCTCAT GCGCCTCAAA AGTGACCTTT TTAATAGGTC CCCGATGTAC CCAGGCCCCA CCAAGGACGA
        90        100        110        120        130        140        150        160
CCCTCTGACC GTGACCTTAG GGTTCACCCT CCAGGACATT GTCAAGGCCG ACTCATCCAC GAACGAGGTG GACCTAGTCT
       170        180        190        200        210        220        230        240
ACTACGAGCA GCAGAGATGG AAGCTCAACA GTCTCATGTG GGATCCAAAC GAGTACGGTA ACATCACCGA CTTCAGGACG
       250        260        270        280        290        300        310        320
TCTGCGGCAG ACATCTGGAC ACCTGACATC ACTGCGTACA GTTCTACAAG ACCTGTACAG GTCCTATCCC CGCAGATTGC
       330        340        350        360        370        380        390        400
TGTTGTCACC CACGACGGCT CTGTTATGTT TATTCCTGCC CAACGCCTCA GCTTCATGTG TGACCCGACT GGGGTAGACA
       410        420        430        440        450        460        470        480
GCGAGGAGGG GGCTACGTGT GCCGTGAAAT TTGGCTCTTG GGTATACAGC GGGTTTGAAA TAGACCTGAA AACGGACACA
       490        500        510        520        530        540        550        560
GACCAAGTGG ATCTCAGCTC GTATTACGCA AGCTCCAAGT ACGAGATTCT GTCAGCTACT CAGACCCGGC AAGTTCAGCA
       570        580        590        600        610        620        630        640
CTACTCATGT TGCCCCGAGC CCTATATAGA TGTCAATCTC GTTGTCAAGT TTCGCGAGAG GCGGGCAGGG AATGGCTTCT
       650        660
TCAGGAATCT CTTTGACTAA
```

(B)

HSQANLMRLKSDLFNRSPMYPGPTKDDPLTVTLGFTLQDIVKADSSTNEVDLVYYEQQRWKLNSLMWDPNEYGNITDFRTS
AADIWTPDITAYSSTRPVQVLSPQIAVVTHDGSVMFIPAQRLSFMCDPTGVDSEEGATCAVKFGSWVYSGFEIDLKTDTDQV
DLSSYYASSKYEILSATQTRQVQHYSCCPEPYIDVNLVVKFRERRAGNGFFRNLFD

S3 Fig. Ac-AChBP DNA and protein sequences. (A) Bacterial codon-optimized DNA sequence (B) Protein sequence used.
