# Supplementary material for: Escherichia coli Protein Expression System for Acetylcholine Binding Proteins (AChBPs)
Source: PLoS One. 2016 Jun 15;11(6):e0157363. doi: 10.1371/journal.pone.0157363 (PMC4909209; doi:10.1371/journal.pone.0157363)
Supplement: S4 Fig — (PDF) [file pone.0157363.s004.pdf]

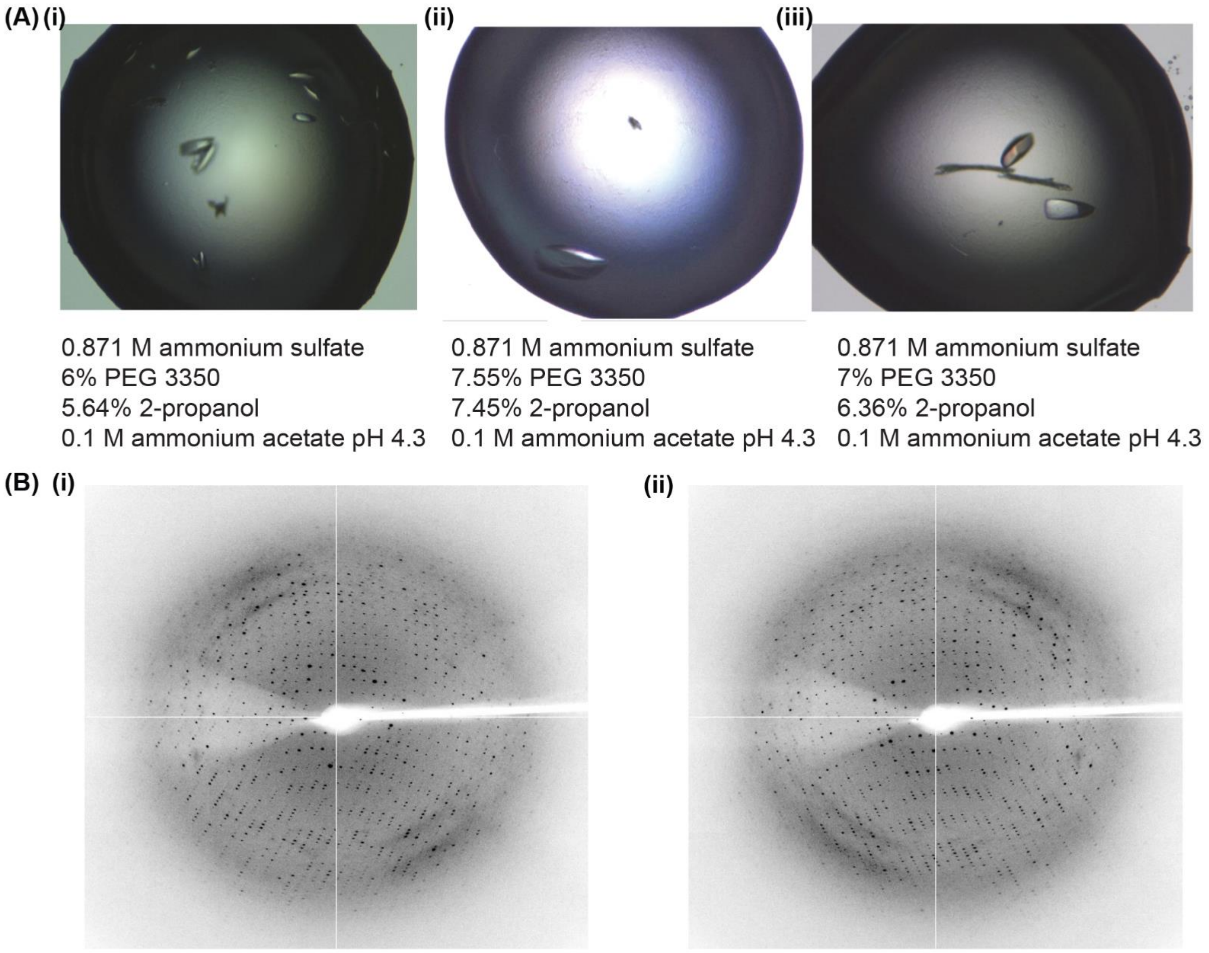

S4 Fig. Crystallization of *E. coli* expressed Ls-AChBP. (A) Single, diffraction quality crystals could be obtained for Ls-AChBP expressed in the *E. coli* expression system. (B) Diffraction pattern for single crystal shown in (A, ii) collected at the MXI beamline at the Australian synchrotron. The first (i) and 180th consecutive image (ii) of a total of 360 images collected at 1° rotation are shown. AChBP crystals diffracted up to 2.8 Å, sufficient for protein structure determination.
