# Supplementary material for: Escherichia coli Protein Expression System for Acetylcholine Binding Proteins (AChBPs)
Source: PLoS One. 2016 Jun 15;11(6):e0157363. doi: 10.1371/journal.pone.0157363 (PMC4909209; doi:10.1371/journal.pone.0157363)
Supplement: S5 Fig — (PDF) [file pone.0157363.s005.pdf]

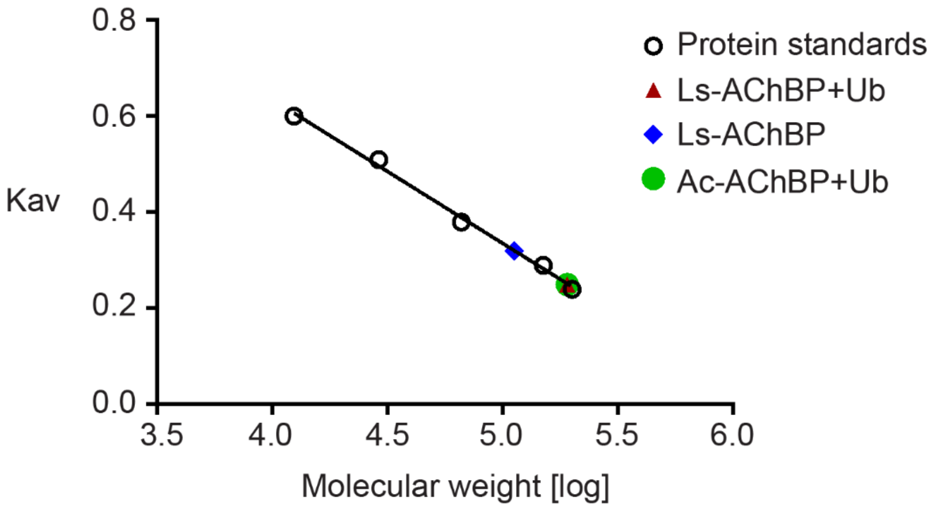

S5 Fig. Calibration curve used to estimate molecular weights for AChBPs. Size-exclusion chromatography column S200 10/300 was calibrated using Blue dextran (2,000,000 Da), β-amylase (200,000 Da), Alcohol dehydrogenase (150,000 Da), Albumin (66,000 Da), Carbonic anhydrase (29,000 Da), and cytochrome C (12,400 Da). The calibration curve was plotted using the gel-phase distribution coefficient (*K*av) versus logarithm of the molecular weight (Log Mw). $K_{av} = (Ve - Vo)/(Vc - Vo)$ where *V*e = elution volume, *V*o = column void volume (7.71 mL based on Blue dextran elution volume), *V*c = geometric column volume (24 mL). Straight line is the calibration curve calculated from the data for molecular weight standards ($R^2 = 0.9955$). Red triangle and green circle correspond to the positions of K_{av} values for Ls-AChBP+Ub and Ac-AChBP+Ub respectively. Blue diamond corresponds to Ls-AChBP. The equation, Y = -0.2993*X + 1.832 from the calibration curve was used to calculate the experimental molecular weights reported in **S1 Table**.
