# Supplementary material for: Escherichia coli Protein Expression System for Acetylcholine Binding Proteins (AChBPs)
Source: PLoS One. 2016 Jun 15;11(6):e0157363. doi: 10.1371/journal.pone.0157363 (PMC4909209; doi:10.1371/journal.pone.0157363)
Supplement: S1 Table — (PDF) [file pone.0157363.s006.pdf]

S1 Table. Experimental and theoretical molecular weights

Protein	Theoretical MW (kDa)	Experimental MW (kDa)
**Ls-AChBP + Ub**	170.9	190.5
**Ls-AChBP**	119.1	112.2
**Ac-AChBP + Ub**	175.9	190.5

*Ac-AChBP was seen to elute as soluble aggregates without Ub and therefore the molecular weight was not calculated. Theoretical molecular weights were calculated using Expasy ProtParam
